# A *MT-TL1* variant identified by whole exome sequencing in an individual with intellectual disability, epilepsy, and spastic tetraparesis

**DOI:** 10.1038/s41431-021-00900-2

**Published:** 2021-06-01

**Authors:** Elke de Boer, Charlotte W. Ockeloen, Leslie Matalonga, Rita Horvath, Solve-RD SNV-indel working group, Enzo Cohen, Isabel Cuesta, Daniel Danis, Anne-Sophie Denommé-Pichon, Yannis Duffourd, Christian Gilissen, Mridul Johari, Steven Laurie, Shuang Li, Leslie Matalonga, Isabelle Nelson, Sophia Peters, Ida Paramonov, Sivakumar Prasanth, Peter Robinson, Karolis Sablauskas, Marco Savarese, Wouter Steyaert, Ana Töpf, Joeri K. van der Velde, Antonio Vitobello, Richard J. Rodenburg, Marieke J. H. Coenen, Mirian Janssen, Dylan Henssen, Christian Gilissen, Wouter Steyaert, Ida Paramonov, Solve-RD-DITF-ITHACA, Siddharth Banka, Elisa Benetti, Giorgio Casari, Andrea Ciolfi, Jill Clayton-Smith, Bruno Dallapiccola, Elke de Boer, Kornelia Ellwanger, Laurence Faivre, Holm Graessner, Tobias B. Haack, Anna Hammarsjö, Marketa Havlovicova, Alexander Hoischen, Anne Hugon, Adam Jackson, Tjitske Kleefstra, Anna Lindstrand, Estrella López-Martín, Milan Macek, Manuela Morleo, Vicenzo Nigro, Ann Nordgren, Maria Pettersson, Michele Pinelli, Simone Pizzi, Manuel Posada, Francesca Clementina Radio, Alessandra Renieri, Caroline Rooryck, Lukas Ryba, Martin Schwarz, Marco Tartaglia, Christel Thauvin, Annalaura Torella, Alain Verloes, Lisenka Vissers, Pavel Votypka, Klea Vyshka, Birte Zurek, Aurélien Trimouille, Tjitske Kleefstra, Alain Verloes, Lisenka E. L. M. Vissers

**Affiliations:** 1 Department of Human Genetics, Donders Institute for Brain, Cognition and Behaviour, Radboud University Medical Center, Nijmegen, The Netherlands; 2 Department of Human Genetics, Radboud University Medical Center, Nijmegen, The Netherlands; 3 CNAG-CRG, Centre for Genomic Regulation (CRG), The Barcelona Institute of Science and Technology, Barcelona, Spain; 4 Department of Clinical Neurosciences, University of Cambridge, John Van Geest Cambridge Centre for Brain Repair, Cambridge, UK; 5 Department of Laboratory Medicine, Translational Metabolic Laboratory, Radboud University Medical Center, Nijmegen, The Netherlands; 6 Department of Human Genetics, Radboud Institute for Health Sciences, Radboud University Medical Center, Nijmegen, The Netherlands; 7 Department of Internal Medicine, Radboud University Medical Center, Nijmegen, The Netherlands; 8 Department of Medical Imaging, Radboud University Medical Center, Nijmegen, The Netherlands; 9 Department of Human Genetics, Radboud Institute for Molecular Life Sciences, Radboud University Medical Center, Nijmegen, The Netherlands; 10 Service de Génétique Médicale, Centre Hospitalier Universitaire de Bordeaux, Bordeaux, France; 11 MRGM, Maladies Rares: Génétique et Métabolisme, lNSERM U1211, Université de Bordeaux, Bordeaux, France; 12 Département de Génétique, APHP Robert DEBRE University Hospital and INSERM U1141, Paris, France; 13 Sorbonne Université, INSERM UMRS_974, Center of Research in Myology, Paris, France; 14 Instituto de Salud Carlos III, Madrid, Spain; 15 Jackson Laboratory for Genomic Medicine, Farmington, CT USA; 16 Inserm - University of Burgundy-Franche Comté, UMR1231 GAD, Dijon, France; 17 Dijon University Hospital, Genetics Department, Dijon, France; 18 Dijon University Hospital, FHU-TRANSLAD, Dijon, France; 19 Radboud Institute for Molecular Life Sciences, Nijmegen, The Netherlands; 20 Folkhälsan Research Center, University of Helsinki, Helsinki, Finland; 21 Department of Genetics, Genomics Coordination Center, University Medical Center Groningen, University of Groningen, Groningen, The Netherlands; 22 Institute of Human Genetics, University of Bonn, Bonn, Germany; 23 MRC Centre for Neuromuscular Diseases and National Hospital for Neurology and Neurosurgery, UCL Queen Square Institute of Neurology, London, UK; 24 John Walton Muscular Dystrophy Research Centre, Translational and Clinical Research Institute, Newcastle University and Newcastle Hospitals NHS Foundation Trust, Newcastle upon Tyne, UK; 25 Manchester Centre for Genomic Medicine, St Mary’s Hospital, Manchester University Hospitals NHS Foundation Trust, Health Innovation Manchester, Manchester, UK; 26 Manchester Centre for Genomic Medicine, Division of Evolution and Genomic Sciences, School of Biological Sciences, Faculty of Biology, Medicine and Health, University of Manchester, Manchester, UK; 27 Med Biotech Hub and Competence Center, Department of Medical Biotechnologies, University of Siena, Siena, Italy; 28 Dipartimento di Medicina di Precisione, Università degli Studi della Campania “Luigi Vanvitelli,”, Napoli, Italy; 29 Telethon Institute of Genetics and Medicine, Pozzuoli, Italy; 30 Genetics and Rare Diseases Research Division, Ospedale Pediatrico Bambino Gesù, IRCCS, Rome, Italy; 31 Donders Institute for Brain, Cognition and Behaviour, Radboud University Medical Center, Nijmegen, The Netherlands; 32 Institute of Medical Genetics and Applied Genomics, University of Tübingen, Tübingen, Germany; 33 Centre for Rare Diseases, University of Tübingen, Tübingen, Germany; 34 Dijon University Hospital, Genetics Department and Centres of Reference for Development disorders and intellectual disabilities, FHU TRANSLAD and GIMI Institute, Dijon, France; 35 Karolinska Institutet, Solna, Sweden; 36 Department of Biology and Medical Genetics, Charles University Prague-2nd Faculty of Medicine and University Hospital Motol, Prague, Czech Republic; 37 Department of Internal Medicine and Radboud Center for Infectious Diseases (RCI), Radboud University Medical Center, Nijmegen, The Netherlands; 38 Department of Genetics, Assistance Publique-Hôpitaux de Paris - Université de Paris, Robert DEBRE University Hospital, 48 bd SERURIER, Paris, France; 39 Institute of Rare Diseases Research, Spanish Undiagnosed Rare Diseases Cases Program (SpainUDP) & Undiagnosed Diseases Network International (UDNI), Instituto de Salud Carlos III, Madrid, Spain; 40 Ospedale Pediatrico Bambino Gesù, Rome, Italy; 41 Medical Genetics, University of Siena, Siena, Italy; 42 Genetica Medica, Azienda Ospedaliero-Universitaria Senese, Siena, Italy; 43 University Bordeaux, MRGM INSERM U1211, CHU de Bordeaux, Service de Génétique Médicale, Bordeaux, France; 44 INSERM UMR 1141 “NeuroDiderot”, Hôpital R DEBRE, Paris, France

**Keywords:** Genetics research, Neurological disorders, Neurodevelopmental disorders, Translational research

## Abstract

The genetic etiology of intellectual disability remains elusive in almost half of all affected individuals. Within the Solve-RD consortium, systematic re-analysis of whole exome sequencing (WES) data from unresolved cases with (syndromic) intellectual disability (*n* = 1,472 probands) was performed. This re-analysis included variant calling of mitochondrial DNA (mtDNA) variants, although mtDNA is not specifically targeted in WES. We identified a functionally relevant mtDNA variant in *MT-TL1* (NC_012920.1:m.3291T > C; NC_012920.1:n.62T > C), at a heteroplasmy level of 22% in whole blood, in a 23-year-old male with severe intellectual disability, epilepsy, episodic headaches with emesis, spastic tetraparesis, brain abnormalities, and feeding difficulties. Targeted validation in blood and urine supported pathogenicity, with heteroplasmy levels of 23% and 58% in index, and 4% and 17% in mother, respectively. Interestingly, not all phenotypic features observed in the index have been previously linked to this *MT-TL1* variant, suggesting either broadening of the m.3291T > C-associated phenotype, or presence of a co-occurring disorder. Hence, our case highlights the importance of underappreciated mtDNA variants identifiable from WES data, especially for cases with atypical mitochondrial phenotypes and their relatives in the maternal line.

## Introduction

The introduction of whole exome sequencing (WES) in clinical settings has massively augmented diagnostic yield for intellectual disability (ID) and other neurodevelopmental disorders (NDD), and additionally identified many new disease-gene associations. Yet, ~50–70% of individuals with ID/NDD remain undiagnosed [1]. The Solve-RD project [2] systematically reanalyzes exomes and phenotypic data of 19,000 unsolved cases with rare disease from four European Reference Networks (ERNs) to elucidate the genetic etiology, including ~5,000 cases from ERN-ITHACA (Intellectual Disability, TeleHealth and Congenital Anomalies; https://ern-ithaca.eu/). Exploration of mitochondrial DNA sequences extracted from WES data is part of this effort [3], as 27 of the 37 mitochondrial genes have a known disease-gene association (http://www.mitomap.org).

*MT-TL1* encodes mitochondrial tRNALeu(UUR), involved in the synthesis of oxidative phosphorylation enzymes by adding leucine to the growing polypeptide chain of mtDNA-encoded subunits during translation [4]. Pathogenic variants in *MT-TL1* have been linked to several phenotypes associated with mitochondrial dysfunction [5], including mitochondrial myopathy, encephalopathy, lactic acidosis and stroke-like episodes (MELAS; MIM#540000) and myoclonic epilepsy associated with ragged-red fibers (MERRF; MIM#545000).

We report on a variant in *MT-TL1* known to interfere with mitochondrial function, uncovered by systematic re-analysis of WES data, illustrating the underexposed potential of WES-based analysis of mtDNA in identifying variants with clinical consequences.

## Methods

### Patient inclusion

All individuals (or legal representatives) in the Solve-RD project provided consent, compliant with local ethical guidelines and the Declaration of Helsinki. For this case, the Radboudumc Ethics Board approved the study (2018-4986). For publication of photos, additional consent was obtained.

### WES

Diagnostic trio-based exome sequencing (proband and parents) was performed as described previously [6] using DNA isolated from whole blood.

### Data sharing

Human Phenotype Ontology-coded clinical data were uploaded along with BAM files to the RD-Connect Genome-Phenome Analysis Platform (https://platform.rd-connect.eu/), and deposited at European Genome-Phenome Archive (EGAZ00001527897), as part of the Solve-RD infrastructure [2]. The variant and phenotype were submitted to the Leiden Open Variation Database (individual number 00328346, phenotype number 0000246573, variant number 0000713909).

### Variant identification

Systematic re-analysis of WES data is described by Matalonga et al. [3]. Details specific to this case include mapping, calling, and annotation of mtDNA using MToolBox pipeline (version 1.0) [7], with mapping against rCRS (for mtDNA) and GRCh37/Hg19 (for genomic DNA) as reference sequences, allowing the detection of heteroplasmy levels and prioritization of variants. The following parameters were applied to identify possible disease-associated variants: (1) coverage ≥8-fold; (2) heteroplasmy fraction ≥1%; (3) GeneBank allele frequency (MITOMAP) <0.2%; (4) “Confirmed” or “Reported” disease association in MITOMAP; and (5) reported “Pathogenic” (ACMG, class 5) or “Likely pathogenic” (ACMG, class 4) in ClinVar.

### Heteroplasmy validation

Confirmation of mitochondrial heteroplasmy was performed on blood and urine of the proband, mother and sisters using routine diagnostic procedures (PGM Ion Torrent Technology).

## Results

### Clinical characteristics

**Fig. 1:**
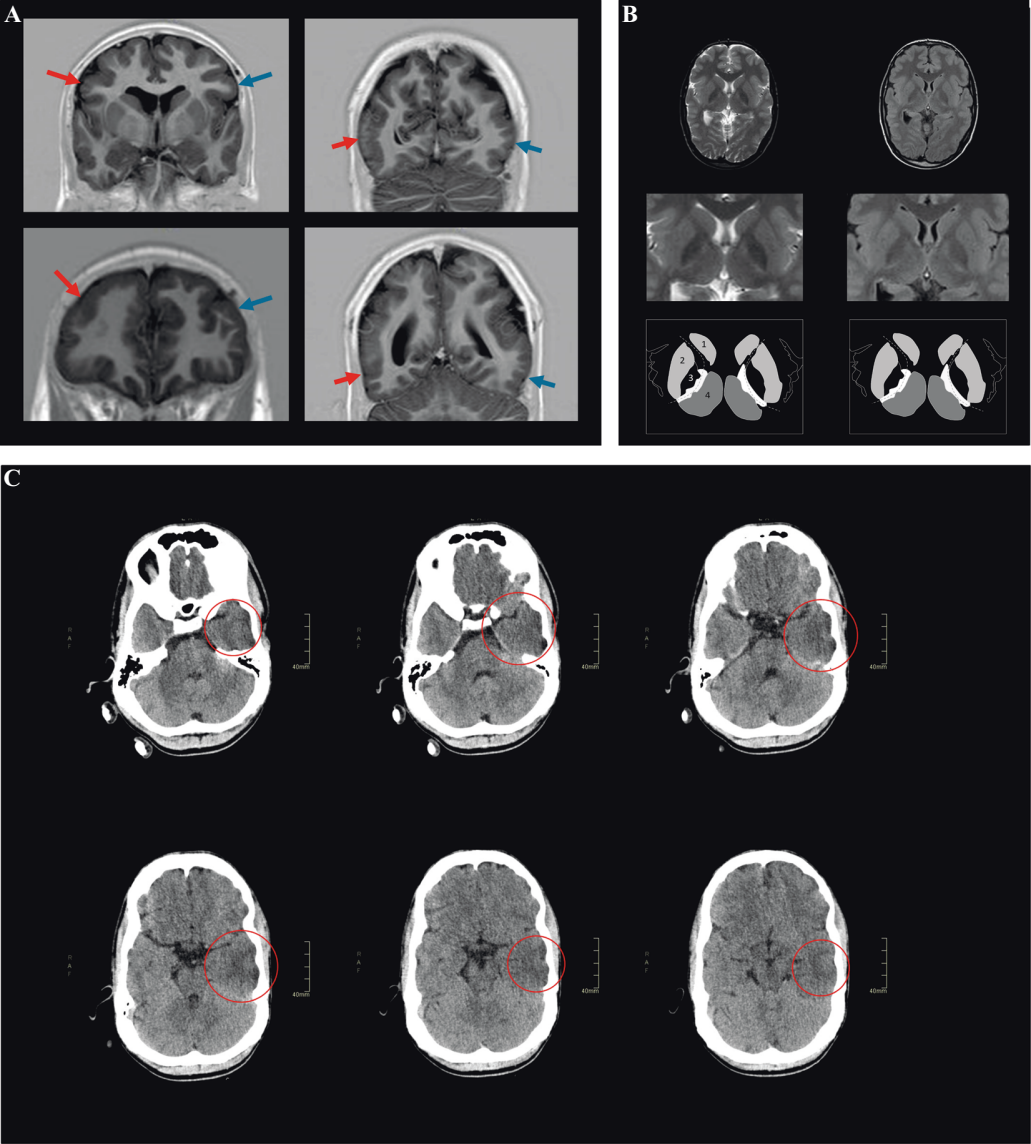
Neuroimaging displays pachygyria, polymicrogyria, white matter abnormalities, and loss of gray–white differentiation. Coronal MRI images (**A**) in the phase-sensitive inversion recovery sequence at age 14 years. The images in the upper row show exemplary regions with a microgyrated aspect (red arrow) as compared to the contralateral region (blue arrow). The images in the lower row show exemplary regions with pachygyration (red arrow). In these regions, the gyrus–sulcus pattern is lost as compared to the contralateral side (blue arrow). Polymicrogyria and pachygyria appear most prominent in the frontoparietal cortical areas. Axial T2-weighted MR images (**B**; left) and fluid attenuation inversion recovery (FLAIR) images (**B**; right) at the level of basal ganglia at age 14 years. The middle row shows a magnification of the basal ganglia derived from images in the upper row, with a schematic representation in the lower row. 1: caudate nucleus; 2: putamen; 3: globus pallidus; 4: thalamus; dotted line: white matter in between the basal ganglia representing the area of the internal capsule. In the posterior limb of the internal capsule, white hyperintensities are present. These can be recognized by their T2-weighted/FLAIR hyperintense aspect and suggest microstructural white matter degeneration. In addition, the globus pallidus on both sides shows a hypo-intense aspect on the T2-weighted images and FLAIR sequence. Axial CT scan images (**C**) in brain tissue setting (W/L: 90/40 HU) at age 17 years. Slides should be viewed from left to right to follow the caudocranial axis; the upper-left corner shows the most caudal slide, the lower-right corner shows the most cranial slide. The red circles indicate the hypodense configuration of the temporal lobe with loss of gray–white differentiation. Gray–white differentiation refers to the appearance of the interface between cerebral white matter and cerebral gray matter on brain CT imaging. Loss of gray–white differentiation often indicates the occurrence of cytotoxic edema. In turn, cytotoxic edema is typical for infarction and hypoxic-ischemic encephalopathic syndromes. A clear asymmetry between the left temporal lobe and the right temporal lobe can be observed. The hypodense configuration involves the superior, middle, and inferior temporal gyri (Color figure online).

We report on a 23-year-old male proband with a complex neurodevelopmental and neuromuscular phenotype, who remained undiagnosed despite extensive diagnostic evaluation in a tertiary center. Family history was unremarkable, with two healthy older sisters and non-consanguineous parents. After uncomplicated pregnancy and delivery at term (normal birth weight (3840 g) and length (52 cm); head circumference 34 cm, 0 SD; Apgar score 10), first concerns about development arose around 3 months of age. At age 15 months, there was severe developmental delay, consisting of hypotonia, delayed motor, social and communicative milestones, and secondary microcephaly (44 cm, −2.5 SD). Brain MRI (at 15 months; repeated at age 14 years) showed supratentorial pachygyria and frontoparietal polymicrogyria (Fig. 1A), with white matter abnormalities in the posterior limb of the internal capsule (Fig. 1B). Cerebellum and corpus callosum showed no deformities and EEG did not show epileptiform activity at age 15 months.

**Fig. 2:**
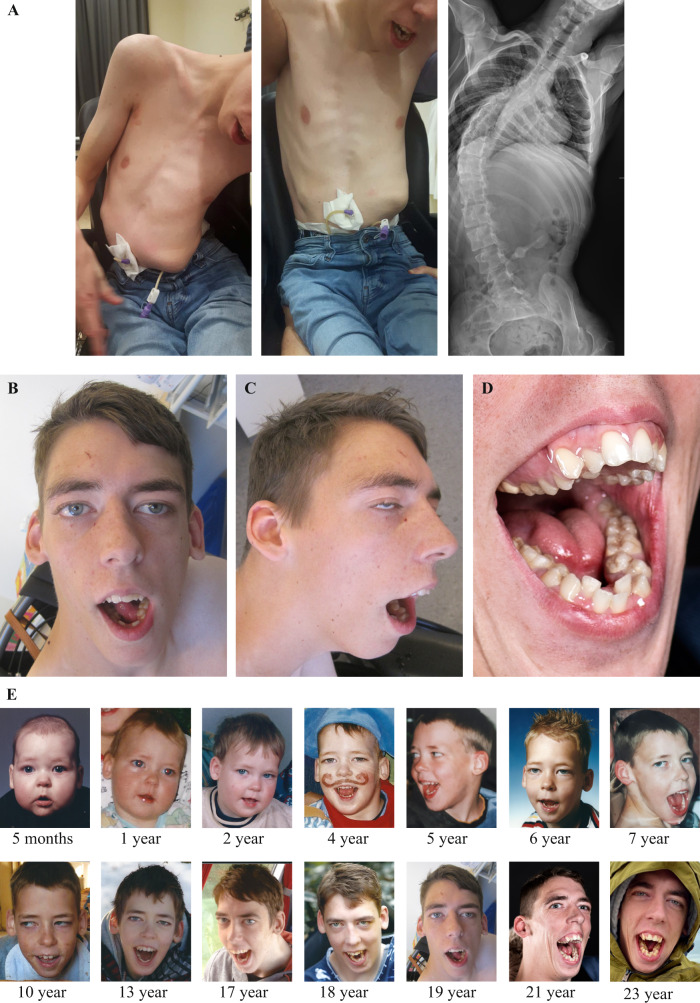
Clinical and radiology images show severe scoliosis, dysmorphisms, and dental crowding. **A** Clinical photographs without traction (left) and with traction (middle) showing asymmetry of the chest and rib protrusion on the left side. Radiograph of the vertebral column (right) with anteroposterior view in supine position shows a slight left convex curvature of the upper thoracic spine and severe right convex scoliosis of thoracolumbar spine (Cobb’s angle ±75°) with axial rotation (asymmetric projection of spinous processes and pedicles) and asymmetry of the thoracic cavity. **B** Frontal and **C** profile facial photographs of the proband (age 19 years), showing a long face with hypotonic appearance, long palpebral fissures, a prominent nose, and small simple ears. The photograph of the mouth (**D**) shows crowded teeth with gingival hyperplasia (age 21 years). Facial photographs between age 5 months and age 23 years (**E**), showing a progression of facial dysmorphisms with advancing age. Only mild dysmorphic features are observed in early childhood, including ptosis and a long philtrum (age 5 months to 4 years). However, the proband develops a progressively pronounced long hypotonic face with open mouth (e.g., photographs at 19 versus 10 versus 2 years of age), and has crowded teeth at age 21 (**D**), whereas teeth appear less crowded at earlier ages (age 7, 13, and 17 years).

He developed spastic tetraparesis, orofacial dystonia, and dystonia of hands and feet. Epilepsy manifested at 7 years of age with frontal focal-onset seizures, with and without secondary tonic-clonic generalization. No myoclonus was observed. Deterioration occurred at age 17 with episodes of severe headaches, accompanied by nausea, vomiting, hematemesis, pallor and perspiration, coinciding with epileptiform activity on EEG in the left temporal lobe. Brain CT was interpreted as normal at that time (Fig. 1C). Different anti-epileptic drugs were prescribed, of which several resulted in adverse drug reactions including erythema multiforme (carbamazepine, oxcarbazepine), muscle weakness (clobazam, pregabalin), or obstructive sleep apnea syndrome (clobazam), resolving after cessation of the respective medication. Because of poor seizure control, 24-h continuous EEG monitoring was performed at 18 years and showed bilateral independent and simultaneous periodic discharges, which were more frequent during sleep than during wakefulness. He is now largely seizure free on low-dose carbamazepine. Other medical problems were severe progressive neuromuscular scoliosis (Fig. 2A), bilateral hip dysplasia with luxation, drooling with excessive tenacious stringy mucus, and severe feeding difficulties requiring gastric tube feeding and resulting in low body weight (age 18: length 165 cm, −2.6 SD; weight 39.5 kg, weight-for-length −3.1 SD; head circumference 54 cm, −1.9 SD). Ophthalmological assessment, visual and brainstem auditory evoked potentials, and electrocardiography were normal. Facial dysmorphisms at 19 years of age included a long hypotonic face, full eyebrows, long palpebral fissures, a prominent nose and nasal bridge, high palate, gingival hyperplasia, and abnormal tooth implant (Fig. 2B–D), becoming more prominent over time (Fig. 2E).

Karyotype, genomic micro-array (Agilent 180k oligo-array), targeted analysis of *WDR62* and *ADGRG1*, extensive lysosomal screening, and trio-based WES did not yield a diagnosis. Metabolic workup revealed elevation of multiple amino acids, including glycine, serine, threonine (in plasma and urine), lysine, methionine, and alanine (in plasma), in a pattern not described for a known metabolic disorder. Lactate in blood (1.8 mmol/l, age 20 years) and cerebrospinal fluid (1.0 mmol/l, age 15 months) were within normal range. Pharmacogenetic analysis showed a rare homozygous variant in *CYP3A4* (NM_017460.5:c.878T > C;*18; rs28371759) unlikely to explain the largely dose-independent adverse drug reactions, and two rare HLA-types (HLA-A*0103; HLA-B*0835) of unknown significance.

### Systematic re-analysis revealed a known pathogenic variant in *MT-TL1*

Data of the proband and parents were included in the Solve-RD project. Prioritization of nuclear DNA variants did not yield diagnostically relevant variants, despite analysis of (*de novo*) variants in known disease–genes, particularly those associated with recessive or dominant cortical dysplasia, and in genes not yet implicated in NDD/ID. Variant prioritization from mtDNA revealed a variant in *MT-TL1* [8], NC_012920.1:m.3291T > C (NC_012920.1:n.62T > C), known to affect mitochondrial function, at 22% heteroplasmy. The variant was absent from maternal WES data.

Validation was performed on blood of the proband and his mother using routine diagnostic procedures, displaying a heteroplasmy level of 23% in the index, compared to 4% in his mother. Urine heteroplasmy levels were 58% and 17% in index and mother, respectively. Follow-up of the family revealed heteroplasmy levels of 4% (blood) and 9% (urine) in one of the sisters, but the variant was absent from blood and urine of the other sister. We re-evaluated brain CT imaging of the index, performed at age 17 years, which in retrospect showed early signs of stroke, including loss of gray–white differentiation in the left temporal lobe (Fig. 1C), co-localizing with epileptiform activity seen on EEG at the time of onset of episodic symptoms. Additionally, multidisciplinary evaluation and comparison of his phenotype to previously published individuals with the same variant (Table 1) revealed that the proband exhibits several symptoms seen in other individuals (epilepsy, episodic headaches with emesis, feeding difficulties, low body weight, and neuromuscular problems) [9–14], but also features that were not described before (abnormalities of brain gyration, facial dysmorphisms, early age of onset of developmental delay).

**Table 1:** Phenotypic comparison of the case presented in this study and those previously published to assess the phenotypic spectrum associated with the *MT-TL1*: m.3291T > C variant (NC_012920.1:m.3291T > C; NC_012920.1:n.62T > C).

Reference	Age of onset (years)	M/F	Phenotype	% heteroplasmy (tissue)	Biochemical–Lactate levels	Other biochemical and/or histochemical evidence	Remarks
Goto et al. [9]	7	M	MELAS; episodic headaches and vomiting, partial and generalized seizures, transient visual and auditory disturbances followed by headaches, cerebral infarctions, mild ID, muscle weakness	86% (muscle), 30% (whole blood), 20% (EBV-transformed lymphocytes)	Elevated (serum and cerebrospinal fluid)	No definite deficiencies of respiratory chain enzymes. RRFs, succinate dehydrogenase reactive vessels	Variant absent in unaffected mother and sister
Uziel et al. [14]	6	F	Mild myopathy: proximal and axial muscle weakness and wasting, normal IQ, impaired growth (weight and length <p3), fatigue	87% (muscle), 45% (lymphocyte), 50% (fibroblast)	Elevated (serum and urine)	Reduction complex I, III, and IV. RRFs and COX-negative fibers, hypotrophic type II fibers, increased lipid content	Mother (below average height, otherwise unaffected) and unaffected brother had heteroplasmy levels of 19% and 6%, respectively
Valente et al. 2009 [18]	51	NA	Myopathy	NA	NA	Normal biochemical parameters. RRFs and COX-negative fibers	
Valente et al. [18]	12	NA	Deafness, cognitive impairment	NA	NA	Reduction complex I. RRFs	
Salsano et al. [19]	Puberty	F	Slowly progressive cognitive decline, behavioral disturbances, Wolff-Parkinson-White syndrome, hearing loss, weight loss, hyperkinesia (myoclonic jerks and tics), cerebellar symptoms and cortical and cerebellar atrophy	95% (muscle), 40% (blood)	Elevated (serum), normal at re-evaluation (serum)	Reduction complex I. RRFs and COX-negative fibers	Variant absent in unaffected mother and sister (blood and urine). Schizophrenia and Wolff-Parkinson-White syndrome are reported for maternal aunts
Sunami et al. [10]	45	F	Severe cerebellar ataxia, myopathy, mild ophthalmoparesis, hearing loss, and asymptomatic EEG abnormality	11% (peripheral leukocytes), 74% (muscle)	Normal (sample not specified)	RRFs, succinate dehydrogenase reactive vessels, COX-negative fibers	Family of 5 affected individuals in 4 generations
Sunami et al. [10]	NA	F	Hearing loss and glaucoma	NA	NA	NA	Family of 5 affected individuals in 4 generations
Sunami et al. [10]	14	F	Palindromic rheumatism, recurrent migraine. Multiple small hyperintense areas in subcortical white matter of cerebrum on T2 MRI	NA	NA	NA	Family of 5 affected individuals in 4 generations
Sunami et al. [10]	36	F	Photo-induced myoclonus, atrophy of cerebellum, absence of tendon reflexes, truncal ataxia, normal mental status	16% (peripheral leukocytes)	NA	NA	Family of 5 affected individuals in 4 generations
Sunami et al. [10]	15	F	Generalized seizures, myoclonic jerks, slight cognitive decline, absence of tendon reflexes	27% (peripheral leukocytes)	NA	NA	Family of 5 affected individuals in 4 generations
Emmanuele et al. [13]	43	M	Progressive myoclonus epilepsy, cerebellar ataxia, cortical and cerebellar atrophy, hearing loss, myopathic weakness, ophthalmoparesis, pigmentary retinopathy, bifascicular heart block, premature graying (20 years)	92% (muscle)	Elevated (serum)	Normal respiratory chain enzymes. RRFs and succinate dehydrogenase reactive vessels	
Yarham et al. [8]	51	M	Bilateral sensorineural deafness, falls, speech disturbance, weight loss, diabetes mellitus, macroglossia with fatty infiltration, dysarthria, bilateral pes cavus, lipoma, low tendon reflexes, dysmetria, generalized brain atrophy	39% (muscle)	Elevated (cerebrospinal fluid)	Dystrophic changes and lipid infiltrates, RRFs, COX-negative fibers, succinate dehydrogenase reactive vessels	Unaffected sister shows heteroplasmy levels of 6% in both urine and blood
Liu et al. [11]	14	F	Progressive cerebellar ataxia, frequent myoclonus seizures, recurrent stroke-like episodes, migraine-like headaches with nausea and vomiting, nystagmus, basal ganglia calcification, brain atrophy, and stroke-like lesions	93% (muscle), 67% (blood), 62% (fibroblasts)	Elevated (serum)	RRF, COX-negative fibers, succinate dehydrogenase reactive vessels	Mother of proband (phenotype: emaciation and short stature) and asymptomatic sister of proband have heteroplasmy levels of 46% and 50%, respectively
Keilland et al. [12]	15	F	Status epilepticus (age 15 years), but in retrospect longstanding history of fatigability, weakness, low body weight (<p3). Ptosis, external ophthalmoplegia, eyelid myoclonia, distal polymyoclonus, stroke-like episodes, gastroparesis, poor gut mobility, constipation, brain abnormalities (old infarction, high signal in parenchym)	75% (muscle), 35% (urine), 30% (blood), 25% (cultured primary skin fibroblast)	Elevated (serum)	Reduction complex I and III. RRFs, succinate dehydrogenase reactive vessels, COX-negative fibers	Mother history of migraine headaches (heteroplasmy in lymphocytes 11%), also headaches in maternal grandmother and maternal great aunt. Brother of proband easily fatigued (heteroplasmy in lymphocytes 14%), sister of proband has headaches (heteroplasmy in lymphocytes 5%)
This publication	23	M	Severe intellectual disability, brain imaging abnormalities (pachygyria, polymicrogyria, white matter abnormalities, in retrospect signs of stroke on brain CT), spastic tetraparesis, oral dystonia and dystonia of hands and feet, epilepsy, episodic headaches with nausea and emesis, adverse drug reactions, feeding difficulties, secondary microcephaly in childhood, low body weight, drooling, severe progressive neuromuscular scoliosis and congenital hip dysplasia, dental and gingival abnormalities, facial dysmorphisms	23% (blood), 58% (urine)	Normal (serum 1.8 mmol/l; age 20 years; and cerebrospinal fluid 1.0 mmol/l, age 15 months)	Not tested	Heteroplasmy levels in the mother: 4% (blood), 17% (urine). The variant was observed in one of the sisters with heteroplasmy levels of 4% (blood) and 9% (urine), but undetectable in blood and urine of the other sister

*COX* cytochrome c oxidase, *ID* intellectual disability, *MELAS* mitochondrial myopathy, encephalopathy, lactic acidosis, and stroke-like episodes, *NA* not available, *RRFs* ragged-red fibers.

## Discussion

Systematic re-analysis of existing WES data of unresolved cohorts can efficiently yield additional diagnoses [15]. Yet, re-analysis rarely includes evaluation of mtDNA although pathogenic mtDNA variants underly many rare diseases. This case illustrates the importance of including mtDNA in re-analysis, as the identification of the *MT-TL1* variant is of medical relevance to the proband and his sisters of childbearing age.

Mitochondrial disorders associated with mitochondrial tRNA genes are characterized by both genotypic and phenotypic heterogeneity, with poor genotype–phenotype correlations [8, 16, 17]. The m.3291T > C variant described here is located in the T-loop of mitochondrial tRNALeu(UUR). This variant was shown to result in respiratory chain enzyme deficiency and its pathogenicity was proven by single muscle fiber mtDNA analysis, showing high heteroplasmy levels in cytochrome c oxidase deficient muscle fibers [8]. Individuals carrying the m.3291T > C variant display a broad phenotypic spectrum varying from mild symptoms to severely debilitating disease [8–14, 18, 19], largely overlapping with features observed in the index. As no additional diagnostically relevant variants could be identified, it remains unclear whether the early age of onset of developmental delay, gyration defects, and dysmorphisms are attributable to a second rare genetic disorder, or expand the m.3291T > C phenotypic spectrum. However, despite gyration defects being uncommon in individuals with *MT-TL1* variants, polymicrogyria has been reported for m.3243A > G in two unrelated individuals with atypical MELAS phenotypes, additionally exhibiting other atypical symptoms also observed in the proband, including hypertonia, early onset developmental delay [20, 21] and facial dysmorphisms [20]. The proband’s facial dysmorphisms might be secondary to muscle tone abnormalities. Hence, we concluded that the *MT-TL1* variant could at least in part, but possibly completely, explain the proband’s phenotype.

In conclusion, we describe a male proband carrying a mtDNA variant confirmed to interfere with mitochondrial function, that was identified in systematic, large-scale re-analysis on a large cohort of individuals with unresolved (syndromic) ID through the collaborative Solve-RD project. Our observations suggest that re-analysis encompassing the mtDNA interpreted from WES data may successfully yield novel unanticipated diagnoses in unexplained cases of ERN-ITHACA with implications for reproductive choices of relatives in the maternal line.

## Supplementary information

Consortium authors
