# Supplementary material for: A MT-TL1 variant identified by whole exome sequencing in an individual with intellectual disability, epilepsy, and spastic tetraparesis
Source: Eur J Hum Genet. 2021 Jun 1;29(9):1359–68. doi: 10.1038/s41431-021-00900-2 (PMC8440635; doi:10.1038/s41431-021-00900-2)
Supplement: Supplementary file 1 — Consortium authors [file 41431_2021_900_MOESM1_ESM.pdf]

SolveRD

Annex I: List of consortium/ groups and corresponding members and affiliations

Solve-RD SNV-indel working group

Enzo Cohen[1], Isabel Cuesta[2], Daniel Danis[3], Anne-Sophie Denommé-Pichon[4,5,6], Yannis Duffourd[4,6], Christian Gilissen[7,8], Mridul Johari[9], Steven Laurie[10], Shuang Li[11], Leslie Matalonga[10], Isabelle Nelson[1], Sophia Peters[12], Ida Paramonov[10], Sivakumar Prasanth[13], Peter Robinson[3], Karolis Sablauskas[7,8], Marco Savarese[9], Wouter Steyaert[7,8], Ana Töpf[14], Joeri K. van der Velde[11], and Antonio Vitobello[4]

Solve-RD-DITF-ITHACA

Siddharth Banka[15,16], Elisa Benetti[17], Giorgio Casari[18,19], Andrea Ciolfi[20], Jill Clayton-Smith[15,16], Bruno Dallapiccola[20], Elke de Boer[7,21], Anne-Sophie Denommé-Pichon[4,5,6], Kornelia Ellwanger[22,23], Laurence Faivre[4,24], Christian Gilissen[7,8], Holm Graessner[22,23], Tobias B. Haack[22], Anna Hammarsjö[25], Marketa Havlovicova[26], Alexander Hoischen[7,8,27], Anne Hugon[28], Adam Jackson[16], Tjitske Kleefstra[7,21], Anna Lindstrand[25], Estrella López-Martín[29], Milan Macek Jr.[26], Leslie Matalonga[10], Manuela Morleo[19], Vicenzo Nigro[19], Ann Nordgren[25], Maria Pettersson[25], Michele Pinelli[19], Simone Pizzi[20], Manuel Posada[29], Francesca Clementina Radio[30], Alessandra Renieri[17,31,32], Caroline Rooryck[33], Lukas Ryba[26], Martin Schwarz[26], Marco Tartaglia[20], Christel Thauvin[4,24], Annalaura Torella[18,19], Aurélien Trimouille[34], Alain Verloes[28,35], Lisenka Vissers[7,21], Antonio Vitobello[4], Pavel Votypka[26], Klea Vyshka[28,35] and Birte Zurek[22,23]

Affiliations

[1]Sorbonne Université, INSERM UMRS_974, Center of Research in Myology, 75013 Paris, France.
[2]Instituto de Salud Carlos III, Madrid, Spain
[3]Jackson Laboratory for Genomic Medicine, Farmington, CT 06032, USA.
[4]Inserm - University of Burgundy-Franche Comté, UMR1231 GAD, Dijon, France.
[5]Dijon University Hospital, Genetics Department, Dijon, France
[6]Dijon University Hospital, FHU-TRANSLAD, Dijon, France.
[7]Department of Human Genetics, Radboud University Medical Center, Nijmegen, The Netherlands.
[8]Radboud Institute for Molecular Life Sciences, Nijmegen, the Netherlands.
[9]Folkhälsan Research Center, University of Helsinki, Finland
[10]CNAG-CRG, Centre for Genomic Regulation (CRG), The Barcelona Institute of Science and Technology, Baldiri Reixac 4, Barcelona 08028, Spain.
[11]Department of Genetics, Genomics Coordination Center, University Medical Center Groningen, University of Groningen, Groningen, The Netherlands.
[12]Institute of Human Genetics, University of Bonn, Bonn, Germany.
[13]MRC Centre for Neuromuscular Diseases and National Hospital for Neurology and Neurosurgery, UCL Queen Square Institute of Neurology, London, UK.
[14]John Walton Muscular Dystrophy Research Centre, Translational and Clinical Research Institute, Newcastle University and Newcastle Hospitals NHS Foundation Trust, Newcastle upon Tyne, UK.
[15]Manchester Centre for Genomic Medicine, St Mary's Hospital, Manchester University Hospitals NHS Foundation Trust, Health Innovation Manchester, Manchester M13 9WL, UK.
[16]Manchester Centre for Genomic Medicine, Division of Evolution and Genomic Sciences, School of Biological Sciences, Faculty of Biology, Medicine and Health, University of Manchester, Manchester, UK.
[17]Med Biotech Hub and Competence Center, Department of Medical Biotechnologies, University of Siena, Italy.
[18]Dipartimento di Medicina di Precisione, Università degli Studi della Campania "Luigi Vanvitelli," Napoli, Italy.
[19]Telethon Institute of Genetics and Medicine, Pozzuoli, Italy.
[20]Genetics and Rare Diseases Research Division, Ospedale Pediatrico Bambino Gesù, IRCCS, 00146 Rome, Italy.
[21]Donders Institute for Brain, Cognition and Behaviour, Radboud University Medical Center, Nijmegen, The Netherlands.
[22]Institute of Medical Genetics and Applied Genomics, University of Tübingen, Tübingen, Germany.
[23]Centre for Rare Diseases, University of Tübingen, Tübingen, Germany.

[24]Dijon University Hospital, Genetics Department and Centres of Reference for Development disorders and intellectual disabilities, FHU TRANSLAD and GIMI InstituteDijon, France.
[25]Karolinska Institutet, Solna, Sweden.
[26]Department of Biology and Medical Genetics, Charles University Prague-2nd Faculty of Medicine and University Hospital Motol, Prague, Czech Republic.
[27]Department of Internal Medicine and Radboud Center for Infectious Diseases (RCI), Radboud University Medical Center, Nijmegen, The Netherlands.
[28]Dept of Genetics, Assistance Publique-Hôpitaux de Paris - Université de Paris, Robert DEBRE University Hospital, 48 bd SERURIER, Paris, France.
[29]Institute of Rare Diseases Research, Spanish Undiagnosed Rare Diseases Cases Program (SpainUDP) & Undiagnosed Diseases Network International (UDNI), Instituto de Salud Carlos III, Madrid, Spain.
[30]Ospedale Pediatrico Bambino Gesù, Rome, Italy.
[31]Medical Genetics, University of Siena, Italy.
[32]Genetica Medica, Azienda Ospedaliero-Universitaria Senese, Italy.
[33]University Bordeaux, MRGM INSERM U1211, CHU de Bordeaux, Service de Génétique Médicale , F-33000 Bordeaux, France.
[34]Service de Génétique Médicale, Centre Hospitalier Universitaire de Bordeaux, Bordeaux, France; MRGM, Maladies Rares: Génétique et Métabolisme, INSERM U1211, Université de Bordeaux, Bordeaux, France.
[35]INSERM UMR 1141 "NeuroDiderot", Hôpital R DEBRE, Paris, France.
